# Dyadic versus individual delivery of a yoga program for family caregivers of glioma patients undergoing radiotherapy: Results of a 3‐arm randomized controlled trial

**DOI:** 10.1002/cam4.5514

**Published:** 2022-12-05

**Authors:** Kathrin Milbury, Meagan Whisenant, Shiao‐Pei Weathers, Smitha Malliaha, Stella Snyder, Natalie Jackson, Jing Li, Yisheng Li, Roseanglea F. Silva, Ya‐Chen Tina Shih, Lorenzo Cohen

**Affiliations:** ^1^ The University of Texas MD Anderson Cancer Center Houston Texas USA; ^2^ The University of Texas Health Science Center at Houston, Cizik School of Nursing Houston Texas USA

**Keywords:** caregivers, dyadic intervention, yoga intervention, primary brain tumors

## Abstract

**Background:**

Despite their significant distress, supportive care interventions for caregivers of glioma patients are generally lacking. And, whether caregivers are more likely to benefit from interventions targeting patient‐caregiver dyads or caregivers individually is unknown. This pilot randomized controlled trial compared the feasibility and preliminary efficacy of a dyadic yoga (DY) versus an individual caregiver yoga (CY) intervention as a supportive care strategy for family caregivers.

**Methods:**

Patient‐caregiver dyads were randomized to a DY, CY or usual care (UC) arm. DY and CY interventions were delivered over 15 sessions. Caregivers completed assessments of their depressive symptoms, quality of life (QOL), and caregiving reactions at baseline, 6 weeks, and 12 weeks, and a subset completed qualitative interviews at 12 weeks.

**Results:**

With a consent rate of 63%, 67 dyads were randomized. Attendance in the DY was higher than in the CY group (session means, DY = 12.23, CY = 9.00; *p* = 0.06). Caregivers (79% female; 78% non‐Hispanic White; mean age, 53 years) reported significantly more subjective benefit in the CY arm than in the DY arm (*d* = 2.1; *p* < .01), which was consistent with the qualitative assessment. There were medium effect sizes for improved mental QOL (*d* = 0.46) and financial burden (*d* = 0.53) in favor of the CY over the UC group. Caregivers in the CY group reported more caregiving esteem (*d* = 0.56) and less health decline (*d* = 0.60) than those in the DY group.

**Conclusion:**

Individual rather than dyadic delivery may be a superior supportive care approach for this vulnerable caregiver population. A larger, adequately powered efficacy trial is warranted.

## Abstract 2

Despite lower intervention session attendance, caregivers in the individual yoga arm reported greater subjective overall benefit, experienced better mental QOL and less caregiver burden compared with those in the patient‐caregiver dyadic yoga and usual care comparison arms. Individual rather than dyadic delivery may be a superior supportive care approach for family caregivers of glioma patients. An adequately powered, larger efficacy trial is warranted.

## 1 INTRODUCTION

Because of the debilitating sequelae, patients with primary brain tumors, particularly those with high‐grade gliomas, often depend on their family members for care and support early in the disease course.^1^, ^2^, ^3^, ^4^, ^5^, ^6^ Although caregiving‐related activities are generally taxing, family caregivers of glioma patients face unique challenges related to patients’ cognitive decline, neurological and motor deficits, and personality changes due to the disease and its treatment.^3^, ^5^, ^6^, ^7^, ^8^, ^9^, ^10^, ^11^, ^12^, ^13^, ^14^, ^15^ In contrast to caregivers of patients with other diseases, caregivers of glioma patients manage both the oncological and neurological concerns of the patients.^16^ Given the typically rapid progression of the disease, caregivers of glioma patients must assist patients with all activities of daily and instrumental living while having to cope with the emotional burden of the uncertainty of the disease course.^1^ Considering the extensive care demands and poor prognosis of glioma patients, it is unsurprising that caregivers of these patients report high rates of psychological distress, fatigue, and sleep disturbances, any of which can undermine the quality of care they are able to provide.^16^, ^17^ In fact, the anxiety and depression levels that caregivers experience may exceed those experienced by glioma patients themselves.^8^

Despite their significant distress, caregivers of glioma patients report that their supportive care needs are often unaddressed by multi‐disciplinary oncology teams.^18^ Moreover, the neuro‐oncology literature generally lacks reports of evidence‐based supportive care interventions for this vulnerable caregiver population.^18^ There are a few exceptions; for instance, one randomized controlled trial showed that cognitive behavioral therapy may increase caregiving mastery and quality of life (QOL) for caregivers of patients with high‐grade gliomas.^19^ In addition, a recent pilot study suggests that an intervention seeking to help caregivers of primary brain tumor patients identify social support networks may improve caregivers’ depressive symptoms.^20^

To address the supportive care needs of both patients and their caregivers, we developed a dyadic yoga intervention that integrates targeted physical exercise with stress‐management techniques to manage symptoms and health‐related QOL.^21^, ^22^ A behavioral intervention that includes both psychological and physical components appears to be promising given the multifaceted needs of families coping with glioma.^23^, ^24^, ^25^ While yoga as a supportive care strategy addressing both physical and psychological/cognitive treatment side effects has been widely studied in patients with cancer (particularly breast cancer), its application to support the wellbeing of their caregivers is limited.^26^, ^27^, ^28^ For caregivers of patients with dementia, a previous trial revealed that yoga significantly improved QOL outcomes.^29^ Whether caregivers are more likely to benefit from a yoga intervention targeting patient‐caregiver dyads or one targeting only caregivers is unknown, as few studies have directly compared the feasibility and efficacy of these types of behavioral interventions. Studies revealing that family caregiver and patient distress and QOL are interdependent seem to support the use of a dyadic intervention, which could create a positive shared experience in the midst of ongoing challenges.^30^, ^31^, ^32^, ^33^, ^34^ However, most dyadic interventions are patient‐focused and enroll caregivers predominately to assist patients or to learn skills to better care for patients. Thus, an individual intervention targeting caregivers’ own symptoms and needs may provide greater benefit to caregivers.

Building on our previous pilot studies, we sought to address these important gaps in the caregiver literature by comparing the feasibility and preliminary efficacy of a patient‐caregiver dyadic yoga (DY) intervention and an individual caregiver yoga (CY) intervention relative to a usual care (UC) group. On the basis of previous trials enrolling caregivers of glioma patients, we hypothesized that at least 50% of eligible dyads would consent to participate; at least 70% of enrolled participants would complete the post‐treatment follow‐up assessment; and that participants would attend at least 2 of every 3 (i.e., 10 of 15) sessions.^19^, ^35^ We also hypothesized that, compared with UC, the CY and DY interventions would have at least medium effect sizes regarding improvements in caregiver depressive symptoms, QOL, and caregiver reactions (i.e., schedule disruption, lack of support, health declines, financial burden, and esteem).

## 2 METHODS

### 2.1 Participants

Patients were asked to identify their primary family caregiver (e.g., spouse/partner, parent, adult child). Both the patient and the caregiver had to be willing to participate in this study and both had to be at least 18 years old; able to read and speak English; and able to provide informed consent. In addition, the patient had to be diagnosed with primary malignant glioma; be scheduled to undergo standard radiotherapy for at least 5 weeks; and have a Karnofsky Performance Status (KPS) score of at least 80 at the time of recruitment. We excluded dyads whose caregiver or patient reported regularly (self‐defined) practicing yoga or whose patient had cognitive deficits that the clinical team deemed would impede the completion of the self‐report instruments.

### 2.2 Procedures

The MD Anderson Institutional Review Board approved all procedures prior to participant enrollment. Research staff used the electronic clinic appointment system to identify potential participants. Before the COVID‐19 pandemic (i.e., prior to March 2020), we approached patients and caregivers during clinic visits and confirmed their eligibility and obtained their written informed consent prior to data collection; if caregivers were not present, we asked patients for their permission to contact their caregivers to obtain consent. During the pandemic (March 2020 onward), all participants were contacted via phone, and consent was obtained electronically. Participants completed survey measures prior to randomization at baseline and completed follow‐up assessments in REDCap at the completion of patients’ radiotherapy (6 weeks) and at the 3‐month follow‐up (12 weeks). To gain further insights into caregivers’ experiences with study participation, a subset of participants (n=15 caregivers) completed individual, qualitative interviews at the 12‐week assessment. (A description of the qualitative interview methods along with participant characteristics is given in the Supplementary S1). Participants received a $20 gift card for each assessment they completed. Although both patients and caregivers completed assessments, the present study focused on caregivers’ outcomes only.

### 2.3 Randomization

After baseline data collection, patient‐caregiver dyads were randomized to the DY, CY, or UC arm through minimization, a form of covariate‐adaptive randomization that ensured that the groups were balanced in terms of patient and caregiver sex and age and patient tumor grade and Karnofsky Performance Status score.^36^

### 2.4 Intervention groups

Participants in the DY and CY intervention groups attended 15 sessions (45 minutes each) over the course of the patients’ 6‐week standard radiotherapy (2 or 3 sessions per week). This dose was selected based on prior yoga efficacy trials that included women with breast cancer undergoing RT where the sessions were provided 2‐3 times a week for 6 weeks.^37^, ^38^ For participants enrolled before the pandemic, sessions 1‐4 were delivered in person at the hospital, and sessions 5‐15 were delivered in‐person or via Zoom, a HIPAA‐compliant videoconferencing platform. For participants enrolled during the COVID‐19 pandemic, all sessions were delivered via Zoom. Three yoga instructors certified by the International Association of Yoga Therapists delivered both interventions.

#### 2.4.1 DY intervention

Patients and caregivers attended DY sessions together. Session content was developed with consideration of the needs and limitations of brain tumor patients as described previously.^39^ Briefly, each DY session included a brief introduction to the session followed by individual and dyadic mind‐body techniques. In accordance with universal Hatha Yoga practices, each session had 4 main components: (a) joint loosening with mindfulness training; (b) postures (asanas) with relaxation techniques; (c) breathing exercises (pranayama); and (d) meditation. The DY sessions highlighted communal coping and interconnectedness within the family, included dyadic postures, and focused the meditations on the family context (e.g., gratitude and lovingkindness mediations were focused on the couple/dyad). Participants were encouraged to offer mutual support during the practice (e.g., stabilizing support during balance poses). Because of contraindications in the patient population, common inversion yoga postures (e.g., standing forward bend, downward‐facing dog) were not used.

#### 2.4.2 CY intervention

The structure and content of the CY sessions were as similar as possible to those of the DY sessions (e.g., no additional poses were included); however, the CY sessions were only attended by the caregiver and did not include a dyadic focus and the partner exercises. Patients in the CY group received UC care as described below. To avoid contamination bias, we asked CY group participants not to share study content with patients for the duration of the study.

#### 2.4.3 Quality control

All yoga sessions were audio‐ and video‐recorded with the participants’ permission, which was obtained during the informed consent process. We reviewed the recordings for treatment fidelity on an ongoing basis using a fidelity checklist.

### 2.5 Control group

Patients in the UC group received usual care from their health care team. Patients and caregivers were invited to attend the monthly Brain Tumor Support Group and had access to a clinical social worker if needed.

### 2.6 Measures

#### 2.6.1 Demographic and medical data

Demographic data (e.g., age, race/ethnicity) were collected with questionnaires at baseline.

Patients’ medical data were extracted from their electronic medical records.

#### 2.6.2 Feasibility data

We documented rates of consent (including refusal reasons), session attendance (including in person and via videoconference), and assessment completion (including attrition reasons). Participants randomized to the DY or CY intervention completed a program evaluation using Likert scale items (see Table 2 for items including scale anchors) that we had developed for our previously published yoga trials.^38^, ^40^

#### 2.6.3 Intervention outcome measures

Depressive symptoms were assessed with the Centers for Epidemiological Studies–Depression (CES‐ D),^41^ which consists of 20 items focused on the affective component of depression. Overall QOL was assessed with the Medical Outcomes Study 36‐item short‐form survey (SF‐36), which has 8 distinct domains (physical functioning, physical impediments to role functioning, pain, general health perceptions, vitality, social functioning, emotional impediments to role functioning, and mental health) and yields both a mental composite summary score and a physical composite summary score.^42^ Caregiving reactions were assessed with the Caregiver Reaction Assessment (CRA), a 24‐item measure assessing four negative caregiving domains (lack of family support, financial strain, schedule disruption, and health problems) and one positive caregiving domain (esteem).^43^

### 2.7 Data analysis

To determine feasibility, we calculated descriptive statistics for consent, session attendance, assessment completion, and program satisfaction. We included the attendance data of all participants who were randomized to the DY and CY arms regardless of their assessment completions. We used *t*‐tests to compare session attendance and program satisfaction between the DY group and CY group. To examine preliminary intervention efficacy for caregivers, we performed multilevel modeling (MLM) using PROC MIXED (SAS, version 9.4). We controlled for the baseline level of the given outcome, included assessment time as a categorical variable, and used CONTRAST statements within the procedure to test for group differences (i.e., DY vs UC, CY vs UC, and DY vs CY). Because the current study was a feasibility study and not adequately powered for definitive efficacy testing, we supplemented the inferential statistics with effect sizes (Cohen's *d*) associated with each between‐group comparison and interpreted each effect size as small (*d* = 0.2), medium (*d* = 0.5), or large (*d* = 0.8).^44^ A medium effect size was considered hypothesis‐confirming.

## 3 RESULTS

### 3.1 Participant characteristics

Patients’ and caregivers’ baseline characteristics by group are shown in Table 1. There were no significant group differences at baseline. Overall, most caregivers were women (79%), were non‐Hispanic White (85%), were spousal caregivers (69%), had some college education (90%; 35% received at least a 4‐year degree), and were employed full‐time (60%). Caregivers had a mean age of 53 years (standard deviation [SD], 11.87 years; range, 30–84 years). Most patients were men (63%), had high‐grade glioma (81%), and had a KPS score of 90 or higher (81%). Patients had a mean age of 48 years (SD, 14.48 years; range, 20–76 years).

**TABLE 1:** Baseline characteristics of patients and caregivers by group

	Patients (*n* = 67)			Caregivers (*n* = 67)		
Characteristic	Dyadic yoga (*n* = 23)	Caregiver yoga (*n* = 22)	Usual care (*n* = 22)	Dyadic yoga (*n* = 23)	Caregiver yoga (*n* = 22)	Usual care (*n* = 22)
Sex, *n* (%)						
Male	16 (70)	12 (55)	14 (64)	4 (17)	4 (18)	6 (27)
Female	7 (30)	10 (45)	8 (36)	19 (83)	18 (82)	16 (73)
Age, years, mean ± SD (range)	49.7 ± 14.3 (10‐74)	48.0 ± 17.8 (35‐84)	48.1 ± 11.2 (33‐75)	53.0 13.7 (30‐81)	53.4 ± 11.2 (35‐84)	51.5 ± 10.4 (33‐75)
Caregiver type, *n* (%)						
Spouse				16 (70)	13 (59)	17 (77)
Other				7 (30)	9 (41)	5 (23)
Race, *n* (%)						
White	21 (91)	19 (86)	22 (100)	20 (87)	19 (86)	22 (95)
Asian	2 (9)	2 (8)	0 (0)	2 (9)	1 (5)	0 (0)
More than one	0 (0)	3 (14)	0 (0)	1 (4)	2 (9)	1 (5)
Non‐Hispanic White, *n* (%)	18 (78)	15 (68)	20 (91)	19 (83)	19 (86)	19 (86)
At least some college, *n* (%)	20 (87)	18 (82)	20 (91)	19 (83)	20 (91)	21 (95)
Household income ≥$75,000, *n* (%)	13 (56)	10 (45)	16 (73)	16 (70)	16 (73)	18 (82)
Employment status, *n* (%)						
Full‐time	7 (31)	5 (22)	12 (55)	9 (39)	11 (50)	9 (41)
Retired	3 (13)	2 (9)	2 (9)	5 (22)	2 (9)	2 (9)
Part‐time	0 (0)	1 (5)	1 (5)	2 (9)	1 (5)	3 (14)
Disability/time off	8 (35)	10 (45)	7 (32)	2 (9)	6 (27)	3 (13)
Home maker	1 (4)	1 (5)	0 (0)	1 (4)	2 (9)	3 (14)
Other	4 (17)	3 (14)	0 (0)	4 (17)	0 (0)	2 (9)
KPS score, *n* (%)						
80	4 (17)	4 (18)	5 (23)			
90	13 (57)	16 (73)	13 (59)			
100	6 (26)	2 (9)	4 (18)			
HGG, *n* (%)	18 (78)	18 (83)	18 (83)			
Lateralization, *n* (%)						
Left	10 (43)	8 (36)	8 (36)			
Right	11 (48)	10 (46)	13 (59)			
Bilateral	2 (9)	4 (18)	1 (5)			
Anticonvulsants, *n* (%)	12 (53)	16 (73)	11 (50)			
Steroids, *n* (%)	10 (43)	11 (50)	11 (50)			
Time since diagnosis, years, mean ± SD (range)	.50 ± 1.1 (.05‐4.2)	.27 ± .41 (.07‐1.9)	.35 ± .62 (.04‐2.5)			
CES‐D, Case^a^, *n* (%)	10 (44)	9 (41)	9 (41)	10 (44)	13 (59)	10 (46)
Support group, *n* (%)				2 (9)	0 (0)	1 (5)
Counseling service, *n* (%)				2 (9)	0 (0)	4 (18)

Abbreviations: CES‐D, Centers for Epidemiological Studies–Depression; HGG, high‐grade glioma; KPS, Karnofsky Performance Status.

^a^ CES‐D score ≥ 16.

### 3.2 Feasibility

#### 3.2.1 Recruitment and retention

**FIGURE 1:**
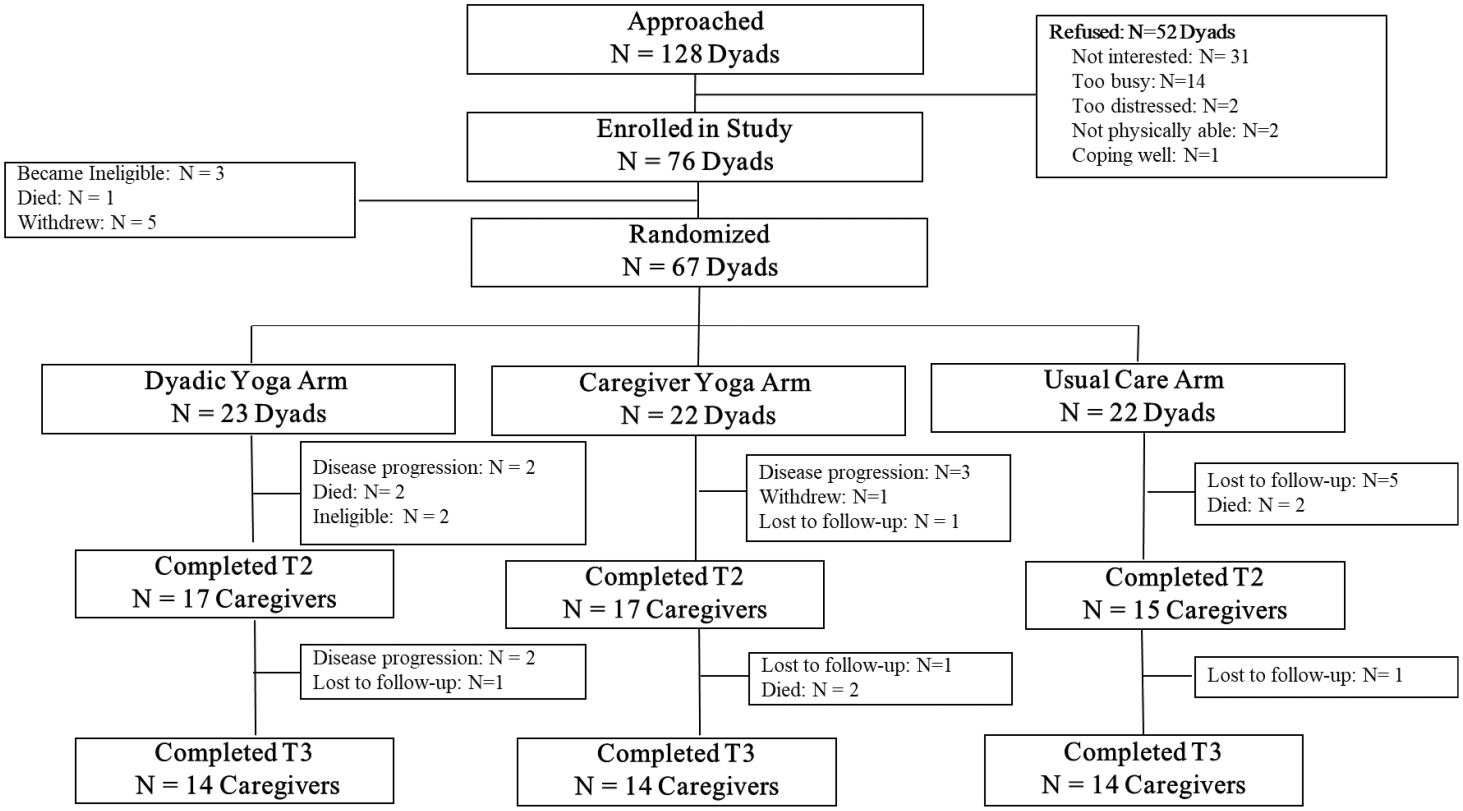
CONSORT flow diagram.

A CONSORT flow diagram illustrating participant recruitment and retention is shown in Figure 1. We approached 128 dyads and obtained consent from 76 (59%). The main reasons dyads declined participation were “not interested” (*n* = 31; 60%) and “too busy” (*n* = 14; 27%). Of the dyads who consented to participation, 3 became ineligible because the patient transferred to hospice, 1 patient died, and 5 dyads withdrew consent prior to randomization so that 67 patient‐caregiver dyads were randomized to receive DY (*n* = 23), CY (*n* = 22), or UC (*n* = 22). Overall, 49 caregivers (73%) completed the 6‐week assessments, and 42 caregivers (63%) completed the 12‐week assessments. Drop‐out was due to high patient symptom burden/disease progression (*n* = 7, 10%), patient death (*n* = 6; 14%), and loss to follow‐up (*n* = 9; 13%), particularly during the pandemic. Attrition was not a function of group assignment (*p* = 0.68); however, patients in the dyads that were not retained had lower KPS scores at baseline (*p* = 0.09).

#### 3.2.2 Session attendance and acceptability

The mean number of sessions the DY participants attended was 12.6 (SD, 4.4); 52% of DY participants attended all 15 sessions, and 81% attended at least 10 sessions. The mean number of sessions the CY participants attended was 10.1 (SD, 5.70); 33% of CY participants attended all 15 sessions, and 62% attended at least 10 sessions. Thus, there was a medium effect (*d* = 0.50) for session attendance in favor of the DY group; however, the group difference was not significant (*t* = 1.6; *p* = 0.11). Owing to the COVID pandemic, 31% of participants (7 dyads in the DY arm and 7 caregivers in the CY arm) participated entirely remotely via Zoom. Prior to the pandemic, only 4 dyads and caregivers chose to attend some sessions (range, 1‐7 sessions) via Zoom. All caregivers in the DY and CY arms recommended the intervention to other caregivers. However, compared with those in the DY arm, caregivers in the CY arm indicated deriving significantly greater benefit from the intervention (*t* = 3.5; *p* = 0.01; *d* = 2.1) and rated the program as more useful (*t* = 3.6; *p* = 0.002; *d* = 1.8). Caregivers in the CY arm also reported having higher expectations regarding the lasting benefit of the intervention than those in the DY arm (*t* = 2.4; *p* = 0.03; *d* = 1.1). The means of the program evaluation variables are given in Table 2. We found a medium effect in favor of in‐person delivery over videoconference delivery (*d* = 0.76), but this effect was not significant (*p* = 0.10). Themes and subthemes from the qualitative interviews are presented in Table 3.

**TABLE 2:** Means of the program evaluation variables

Variable	DY group *N* = 23	CY group *N* = 22	
	Mean ± SD (range)	Mean ± SD (range)	*d*
Overall program usefulness^a^	2.2 ± .42 (2‐3)	2.9 ± .4 (2‐3)	1.8*
2 Borg Exertion Scale^b^	2.6 ± .84 (1‐4)	3.0 ± .54 (2‐4)	.57
3 Subjective overall benefit of program (mean score)^c^	4.4 ± .53 (3.64‐5.29)	5.4 ± .4 (4.79‐6.00)	2.1**
Calm your mind	5.1 ± .99 (4‐6)	5.8 ± .5 (5‐6)	.78
b Increase your emotional wellbeing	4.6 ± .92 (3‐6)	5.6 ±.6 (5‐6)	1.3*
c Increase your flexibility	4.4 ± 1.07 (3‐6)	5.8 ± .5 (5‐6)	1.7*
d Increase your mental focus	4.5 ± .54 (4‐5)	5.4 ± .9 (4‐6)	1.2*
e Increase your mobility	3.9 ± .84 (3‐5)	5.4 ± .9 (4‐6)	1.8*
f Improve your mood	5.0 ± .76 (4‐6)	5.6 ± .6 (5‐6)	.90
g Increase your physical energy	3.9 ± 1.23 (2‐5)	5.0 ± .7 (4‐6)	1.1*
h Increase your physical wellbeing	4.1 ± .64 (3‐5)	5.0 ± .7 (4‐6)	1.3*
i Improve your posture and balance	4.3 ± .87 (3‐5)	5.2 ± .8 (4‐6)	1.11*
j Present more aware of the present moment	4.9 ± .64 (4‐6)	5.0 ± 1.0 (4‐6)	.14
k Improve range of motion	4.0 ± .76 (3‐5)	5.6 ± .9 (4‐6)	1.9**
l Relax your body	5.1 ± 1.13 (3‐6)	6.0 ± .0 (6)	1.1
m Improve your social relationships with others	3.4 ± 1.19 (1‐5)	4.6 ± 1.4 (3‐6)	.96
n Increase sense of emotional stability	4.5 ± .93 (3‐6)	5.6 ± .6 (5‐6)	1.4*
4 Expectation of lasting benefit (mean score)^d^	4.2 ± .4 (3.7‐4.7)	4.6 ± .4 (3.9‐5.0)	1.1*

*Note*: Cohen's *d* was interpreted as follows: *d* = 0.2, small effect; *d* = 0.5, medium effect; and *d* = 0.8, large effect. (*p < 0.05, **p < 0.01; *t*‐test).

Abbreviations: CY, caregiver yoga; DY, dyadic yoga; ES, effect size; SD, standard deviation.

^a^ Scores ranged from 1 (“not at all”) to 3 (“very much”).

^b^ Scores ranged from 0 (“rest”) to 10 (“maximal; just like my hardest race).

^c^ Scores ranged from 0 (“not at all”) to 6 (“very much”).

^d^ Scores ranged from 1 (“strongly disagree”) to 5 (“strongly agree”).

**TABLE 3:** Quotes from caregivers illustrating themes related to feasibility and acceptability of the yoga intervention

Theme	Quotes
Theme category: yoga was beneficial for the caregiver	
Yoga gave the caregiver something to do for self	Yoga was something I needed to do to take care of myself at the same time as I was taking care of [the patient]. *Female, 43 yrs old, caring for father, DY263* As an old guy, yoga was good because it helped me stretch, and it gave me something to do for me. *Male, 61 yrs old, caring for son, DY276*
Yoga helped the caregiver relax (caregiver yoga arm only)	I have done yoga on and off for years. I was not in a program and to get back to that and get the calming benefits of it was wonderful for me. It is a lot about the breathing, and the breathing makes you center, and the center makes you calmer. *Female, 66 yrs old, caring for daughter, CY270* I think it is just the overall feeling of relaxation and calm and spiritual connection in probably the most challenging time of my life. *Female, 44 yrs old, caring for mother, CY266*
Yoga gave the caregiver something to do with the patient (dyadic yoga arm only)	Especially right now with the pandemic, we are not allowed to go with [the patient] anywhere, to the appointments or anything. It was something for me to be included in with him and not whooshed out or have to stay outside for. *Female, 73 yrs old, caring for spouse, DY273* I think just this time that I spent with [the patient] was important. *Female, 43 yrs old, caring for father, DY263*
Theme category: yoga was beneficial for the patient	
Yoga helped the patient relax (dyadic yoga arm only)	Sometimes [the patient] would get anxious or overwhelmed. And I think with the yoga, it helped a lot to be able to find techniques to just relax. We did use it a lot at night to unwind and just be able to sleep. *Female, 43 yrs old, caring for father, DY263* I know yoga helped [the patient] relax because when we would start doing the relaxing techniques, he would fall asleep. So that might be the only time he would sleep. *Female, 65 yrs old, caring for spouse, DY257*
Yoga helped the patient with movement and activity (dyadic yoga arm only)	Yoga helped [the patient] get in and out of the bathtub; it seemed to loosen his muscles and gave him more flexibility. *Female, 65 yrs old, caring for spouse, DY257* Yoga just gave us time to focus on our bodies and movement. *Female, 34 yrs old, caring for spouse, DY277*
Theme category: yoga was beneficial for dyadic relationship	
Yoga gave the dyad something to look forward to together (dyadic yoga arm only)	I really liked yoga because it was really good for us to do it together; it gave us something to do. We looked forward to it. *Female, 34 yrs old, caring for spouse, DY277* I would say yoga was a bright star because it gave us something to look forward to. *Male, 61 yrs old, caring for son, DY276*
Yoga helped the dyad focus beyond the disease and treatment (dyadic yoga arm only)	Yoga is something to take our mind off of what else is going on, and I was really thankful for that time. It made a long‐drawn‐out situation a little bit better. *Female, 73 yrs old, caring for spouse, DY273* I was thankful for just the pause in the day. Yoga ended up being almost like a retreat for me in a lot of ways just to help take my mind off of things. The yoga did help in that it broke the day and introduced another person into our life that kind of helped us have a different thought pattern. *Female, 43 yrs old, caring for spouse, DY273*
Theme category: acceptability of caregiver‐only yoga intervention	
Yoga helped caregiver find respite from caregiving responsibilities (caregiver yoga arm only)	Yoga gave me some time to get away. It forced me to take time off and just relax. *Female, 44 yrs old, caring for mother, CY266* Yoga was an absolutely wonderful escape from the situation and it helped me significantly get through it all. It gave me an outlet to be able to do that for forty‐five minutes every day. *Female, 46 yrs old, caring for sister, CY271*
Yoga may be helpful for the patient (caregiver yoga only arm)	I think doing yoga would be really good for her. It was for me. *Female, 60 yrs old, caring for daughter, CY264* I would go back and do yoga more with the patient and the caregiver. I think that would be a good connection for the two, and I think it would benefit the patient. I thought the yoga program was wonderful. I thought it was a great way for the hospital to reach out past the patient. But I do think it needs to be patient and caregiver if both feel up to it and are able to do it. *Female, 66 yrs old, caring for daughter, CY270*
Theme category: acceptability of virtual yoga intervention	
Virtual setting was convenient for caregivers participating in the yoga intervention	It was actually easier because I didn’t have to leave work early or anything like that. So that was good. I was even able to do it while on vacation, so that worked as well. It wasn’t challenging at all. *Female, 44 yrs old, caring for mother, CY266* I liked the fact that it was remote because I didn’t have to spend time going somewhere, leaving somewhere, or being away from the patient. *Female, 46 yrs old, caring for sister, CY271*
Caregivers may have enjoyed an in‐person yoga intervention	I wish we would have been able to meet in person and actually be in front of the instructor. But it was a good experience overall. *Female, 43 yrs old, caring for father, DY263* We did yoga by video; maybe if I would have been able to do it in person, that might have been a little better, but I don't think I missed anything by doing it through a Zoom meeting. *Female, 60 yrs old, caring for daughter, CY264*
Theme category: continued use of yoga following study participation	
Continuing to practice yoga after study participation	And even now sometimes, when [the patient] is on chemo and he is not feeling well, or he is just feeling anxious, we will do some of the relaxation yoga that we have available still. *Female, 43 yrs old, caring for father, DY263* Well, we have continued that. I mean, we both stretch. We stretch each other now. We are doing a little bit of that before, but we do a lot of things. But he has gotten so much better. I mean, it is hard to point out what he was able to do then and what he can do now. It is like night and day. *Male, 61 yrs old, caring for son, DY276*
Challenges in making time for yoga after study participation	I think that it would be great if I had the time. I did yoga as much as I could. I don't have the time, but I think it would be a good way to refocus and not be so anxious. *Female, 59 yrs old, caring for spouse, CY278* I intended on doing yoga when I left Houston. I mean, I really did, and we got back home I did not make time for it and have not made time for it. I have a lot of issues with pain in my hip and if I could have kept it up, it might have helped with some of that. *Female, 34 yrs old, caring for spouse, DY277*

### 3.3 Preliminary efficacy

The raw means of the outcome measures at each assessment point by group are given in Table 4. Least square means (LSMs) from the MLM analyses (controlling for baseline level of the outcome and assessment point) are presented below.

**TABLE 4:** Raw means and standard deviations (SDs) of outcome measures at each assessment point by group

	Mean (SD) at baseline			Mean (SD) at 6 weeks			Mean (SD) at 12 weeks		
Measure	DY	CY	UC	DY	CY	UC	DY	CY	UC
CES‐D	14.2 (9.1)	17.0 (9.4)	15.9 (10.7)	12.1 (9.2)	14.1 (10.7)	12.0 (8.9)	9.6 (7.4)	7.9 (5.6)	9.8 (7.2)
SF‐36 MCS	46.2 (11.9)	45.0 (10.0)	46.7 (9.8)	45.5 (14.0)	47.3 (11.4)	47.2 (13.0)	41.2 (6.0)	46.5 (2.8)	44.1 (5.4)
SF‐36 PCS	43.8 (6.2)	45.8 (4.6)	41.7 (5.6)	41.6 (8.5)	45.6 (3.9)	42.5 (9.5)	51.9 (11.3)	48.7 (9.4)	47.9 (10.8)
CRA Health	1.9 (0.7)	1.9 (0.9)	2.1 (0.6)	2.0 (0.6)	1.8 (0.7)	1.8 (0.7)	2.0 (0.6)	1.6 (0.5)	1.8 (0.8)
CRA Finance	2.6 (1.1)	2.5 (1.2)	2.2 (1.1)	2.7 (1.0)	2.5 (1.2)	2.4 (1.4)	2.5 (0.9)	1.9 (0.8)	2.4 (1.2)
CRA Schedule	3.5 (0.7)	3.6 (0.7)	3.4 (1.0)	3.3 (0.8)	3.4 (0.8)	3.4 (1.0)	3.3 (0.8)	2.9 (0.9)	2.8 (1.1)
CRA Support	1.7 (0.7)	1.7 (0.7)	1.7 (0.9)	1.6 (0.5)	2.0 (0.6)	1.6 (0.9)	1.8 (0.5)	1.7 (0.7)	1.8 (1.0)
CRA Esteem	4.6 (0.5)	4.8 (0.4)	4.6 (0.6)	4.4 (0.5)	4.7 (0.4)	4.8 (0.4)	4.5 (0.6)	4.8 (0.4)	4.6 (0.4)

Abbreviations: CES‐D, Center for Epidemiologic Studies–Depression; CRA Esteem, CRA Esteem Subscale; CRA Financial, CRA Financial Problems Subscale; CRA Health, Caregiver Reaction Assessment (CRA) Health Subscale; CRA Schedule, CRA Disrupted Schedule Subscale; CRA Support, CRA Lack of Family Support Subscale; CY, caregiver yoga group; DY, dyadic yoga group; MCS, mental component score; PCS, physical component score; SF‐36, Medical Outcomes Study 36‐item short‐form survey; UC, usual care group.

#### 3.3.1 Depressive symptoms

The MLM analyses revealed no group differences in CES‐D scores (LSMs: CY, 11.6; DY, 10.6; UC, 11.4).

#### 3.3.2 Mental and physical QOL

Planned comparisons within the MLM analyses revealed a medium effect (*d* = 0.46) between the CY and UC group for the SF‐36 mental composite summary (*F* = 2.4; *p* = 0.13; LSMs: CY, 49.8; DY, 47.3; UC, 46.3; [higher scores indicate higher QOL]). There was no effect between the DY and UC groups (*d* = 0.13) and a small effect between the CY and DY groups (*d* = 0.33) in favor of the CY group. There were no group differences in the SF‐36 physical composite summary (LSMs: CY, 43.2; DY, 43.5; UC, 44.2).

#### 3.3.3 Caregiver reactions

Planned comparisons revealed marginally significant differences in the health, finance, and esteem subscales of the CRA among the 3 groups. Specifically, for caregiving‐related health declines, although the effect between the CY and UC groups was small (*d* = 0.23), the difference between the CY and DY groups was significant (*F* = 3.9; *p* = 0.05; *d* = 0.60), with caregivers in the CY group reporting less burden (LSMs: CY, 1.7; DY, 2.0; UC, 1.8 [lower scores indicate lower burden]). The effect between the DY and UC groups was also small (*d* = –0.38) but in the opposite direction. There was a marginally significant difference in financial burden related to caregiving between the CY and UC groups (*F* = 3.1; *p* = 0.09) revealing a medium effect (*d* = 0.53) in favor of the CY intervention (LSMs: CY, 2.2; DY, 2.4; UC, 2.6). The effects between the DY and UC groups (*d* = 0.30) and the CY and DY groups (*d* = 0.22) were small. There were no group differences in the schedule disruption and lack of family support subscales. There were marginally significant differences in the positive domain of the CRA, with medium effect sizes, between the DY and UC groups (*F* = 2.9; *p* = .09; *d* = 0.50) and between the DY and CY groups (*F* = 3.7; *p* = 0.06; *d* = 0.56; LSMs: CY, 4.7; DY, 4.5; UC, 4.7 [higher scores indicate greater esteem]), suggesting that caregivers in the DY group derived *less* esteem from their caregiving role than did those in the CY and UC groups.

## 4 DISCUSSION

The goal of this pilot randomized controlled trial was to assess the feasibility and preliminary efficacy of a yoga intervention for caregivers of glioma patients undergoing radiotherapy that was delivered to either patient‐caregiver dyads or caregivers individually. Our results revealed that the intervention is feasible, as our a priori criteria regarding consent, retention, and attendance were met in both the CY and DY groups. Although the DY group had a higher numeric attendance rate, both the DY and CY groups reached the a priori attendance rate benchmark of 67%. It appears that delivering the interventions concurrently to patients’ radiation treatment plan facilitates session attendance regardless of modality (i.e., in‐person at the hospital or via videoconference). The overall 3‐month follow‐up retention rate was relatively low (62%), and attrition was mainly due to patients’ high symptom burden, disease progression, and death—a challenge that is frequently encountered in studies that include caregivers of patients whose expected survival time is short. Moreover, with the onset of the COVID‐19 pandemic, caregivers’ access to the hospital was restricted, and participants received follow‐up care from their medical team mainly via telemedicine. Thus, attrition may have also been a result of less frequent clinic visits.

We were particularly interested in determining whether caregivers favor and benefit dyadic or individual delivery of the intervention. Although dyadic delivery as a care model has increasingly gained attention in psycho‐oncology and supportive care, our results revealed that caregivers in the CY arm found the intervention significantly more useful and beneficial than those in the DY arm. These quantitative results are supported by the qualitative accounts highlighting that caregivers in the CY arm enjoyed practicing self‐care and having time to relax and escape their caregiving role. Although caregivers in the DY arm mentioned benefits to the patient and benefits to their relationship with the patient, they did not mention the benefits of respite and relaxation. The dyadic setting may not allow caregivers to fully relax and focus on themselves as they remain aware of the needs of the patient. This finding may be particularly true for those caring for patients with cognitive deficits. Lastly, although the transition to videoconference delivery with the onset of the pandemic did not impact attendance, our effect size results indicate that caregivers favored in‐person delivery. However, caregivers also acknowledged the benefits of remote delivery in the qualitative interviews.

Our findings also suggest that, even with the lower attendance rate of the CY group, the CY intervention revealed stronger evidence for preliminary efficacy than the DY intervention in terms of improved mental QOL and caregiver reactions. Although the trial was not adequately powered to definitively assess efficacy, our hypotheses were supported by the medium effect sizes for improved mental QOL and financial burden, the latter of which could be driven by the larger proportion (albeit not statistically significant) of caregivers in the CY group who were employed full‐time. As such, group differences regarding financial burden need to be interpreted with caution particularly as the intervention did not target financial distress. Yet, as the subscale assessing financial burden measures *perceptions* of financial distress rather than objective financial toxicity/hardship, aspects of the intervention such as gratitude and lovingkindness meditations may alter participants’ perceptions of financial burden. Further investigations are needed to corroborate our findings.

Moreover, we would like to note that there was not only a lack of evidence regarding the benefits for the DY group relative to the UC group, caregivers in the DY group also reported *worse* outcomes for the esteem and health decline subscales of the CRA. We found no evidence of preliminary efficacy for depressive symptoms and physical QOL in either the DY or CY group, which is surprising considering that caregivers, particularly those in the CY arm, reported subjective emotional, cognitive, and physical wellbeing benefits on the program evaluations. However, the caregivers were experiencing low levels of depressive symptoms at study entry, with only 49% meeting the minimum threshold for clinical screening criteria for depression, suggesting a potential floor effect may have influenced study findings. And, the relatively low assessment completion rates, even among participants with high intervention attendance (i.e., ≥10 sessions), may have contributed to a failure to capture all intervention effects.

Our findings generally agree with those of the few previous studies in this population. For instance, a Dutch study found that a 6‐session, psychologist‐led cognitive behavioral therapy intervention had a small effect on mental QOL.^19^ In addition, a recent study showed that a cognitive behavioral therapy–based intervention had a medium effect on caregiving‐specific distress but failed to yield significant group differences for depressive symptoms.^35^ Another study showed that an electronic social network intervention may improve depressive symptoms but may not have an effect on caregiver burden or other psychosocial outcomes.^20^

Given the findings of the present study, further investigation of the CY intervention is warranted. Our findings suggest that, for caregivers of patients with primary brain tumors, a caregiver‐focused supportive care delivery is preferable to dyadic approaches. However, further study that includes the direct comparisons of dyadic and individual delivery is needed along with models that more precisely establish settings in which a dyadic, individual or a combination of dyadic and individual components as in a “hybrid” (or even group‐based) format is advantageous should be developed. For instance, caregivers of patients who have early‐stage disease and/or less disease burden may respond to dyadic interventions, whereas those facing a high caregiving load may benefit from individual interventions.

The findings of the present study should be interpreted with caution. The objective of this trial was to examine the feasibility and *preliminary* efficacy of the DY and CY interventions. The trial was not designed to confirm efficacy, as it was underpowered. The a priori benchmark regarding a medium effect size was rather conservative; however, its use is justifiable, as we did not control for non‐specific intervention effects, such as attention, in the UC group. Moreover, our study is limited by the participants’ fairly homogenous characteristics, particularly race/ethnicity and socio‐economic status.

In summary, the findings of the present study suggest that, although both the CY and DY interventions are feasible, the CY intervention is subjectively more beneficial to caregivers of glioma patients. This study also revealed that the use of a videoconferencing platform (Zoom in this case) is an acceptable approach for the delivery of interventions to caregivers of glioma patients. Finally, the findings of this trial suggest that relative to the DY intervention and UC, the CY intervention has greater preliminary efficacy in terms of improving mental health aspects of QOL as well as caregiving‐related esteem, health decline, and financial burden. An adequately powered efficacy trial of the CY intervention with an attention control group is warranted.

## AUTHOR CONTRIBUTIONS

**Kathrin Milbury:** Conceptualization (lead); data curation (lead); formal analysis (equal); funding acquisition (lead); investigation (lead); methodology (lead); project administration (lead); resources (lead); supervision (lead); writing – original draft (lead). **Meagan Whisenant:** Conceptualization (equal); data curation (equal); formal analysis (equal); methodology (supporting); project administration (supporting); writing – original draft (supporting); writing – review and editing (equal). **Shiao‐Pei S Weathers:** Conceptualization (equal); funding acquisition (equal); methodology (equal); project administration (supporting); writing – review and editing (equal). **Smitha Mallaiah:** Conceptualization (supporting); data curation (supporting); investigation (supporting); methodology (supporting); project administration (supporting); writing – review and editing (supporting). **Stella Snyder:** Data curation (equal); investigation (supporting); project administration (supporting); supervision (supporting); writing – review and editing (supporting). **Natalie Jackson:** Data curation (supporting); formal analysis (supporting); writing – review and editing (supporting). **Jing Li:** Conceptualization (supporting); data curation (supporting); funding acquisition (supporting); investigation (supporting); writing – review and editing (supporting). **Yisheng Li:** Conceptualization (equal); formal analysis (lead); methodology (equal); project administration (supporting); writing – review and editing (supporting). **Rosangela Fatima Silva:** Data curation (supporting); project administration (supporting); writing – review and editing (supporting). **Ya Chen Tina Shih:** Conceptualization (equal); funding acquisition (equal); investigation (supporting); methodology (equal); writing – review and editing (supporting). **Lorenzo Cohen:** Conceptualization (equal); funding acquisition (supporting); investigation (supporting); methodology (equal); resources (supporting); writing – review and editing (supporting).

## CONFLICT OF INTEREST

None.

## ETHICS STATEMENT

The MD Anderson Institutional Review Board approved all procedures prior to participant enrollment.

## TRIAL REGISTRATION

ClinicalTrials.gov NCT02481349

## Supporting information


Supplementary S1.

## Data Availability

The data that support the findings of this study are available from the corresponding author upon reasonable request.
