# Supplementary material for: Dyadic versus individual delivery of a yoga program for family caregivers of glioma patients undergoing radiotherapy: Results of a 3‐arm randomized controlled trial
Source: Cancer Med. 2022 Dec 5;12(6):7567–79. doi: 10.1002/cam4.5514 (PMC10067051; doi:10.1002/cam4.5514)
Supplement: Supplementary file 1 — Supplementary S1. [file CAM4-12-7567-s001.docx]

**SUPPLEMENTAL MATERIALS**

**Qualitative** **Methods**

To gain a broad understanding of patients’ and caregivers’ experiences in the different study groups, we conducted individual semi-structured interviews with patients and caregivers at the T3 assessment point (as an optional procedure obtained during the consent process). Sampling for qualitative interviews continued to thematic saturation, with no new themes identified in three sequential interviews. Individual interviews (30-45 minutes) were conducted by telephone by a nurse-scientist (MW) with extensive experience in qualitative interviewing following an interview guide. At the end of each interview, the interviewer recorded a field note of the circumstances and events during the interview. All interviews were digitally recorded and professionally transcribed for analysis. Here, we report on qualitative data pertaining to study participation and feasibility from qualitative interviews with 11 caregivers (5 DY caregivers, 6 CY caregivers). Additional themes from patient (n=11) and caregiver (n=15) interviews across all three study arms will be described in a subsequent manuscript.

**Data Analysis Plan for Qualitative Findings**

We conducted qualitative content analysis of semi-structured interviews using MAXQDA2022 (VERBI GmbH, Berlin, German) qualitative analysis software. After the interviews were completed for each arm, an initial coding of themes pertaining to evaluate the feasibility and acceptability of the yoga interventions was conducted. The analysis team (MW, NJ, and KM) met and reviewed the coding. Changes were made to the coding until team consensus was reached. A final description of themes of the patient and caregiver experience of the yoga interventions were developed and compared across the DY and CY arms.

**Demographic Characteristics of Interviewed Caregivers**

Of the 11 interviewed caregivers (5 in the DY and 6 in the CY group), 10 (91%) were female; 9 (82%) were non-Hispanic White; and 7 (64%) were non-spousal family caregivers caring for a male patient (64%) with a KPS of 90 (64%). Caregivers had an average age of 56 years (SD=12 years; range= 34-73 years); 7 (64%) had at least a college degree; and 9 (82%) had a combined household income over $50,000. Of those interviewed, 9 (82%) caregivers received the intervention via videoconference.
